# Combining dance movement therapy and pharmacotherapy in adolescents with major depressive disorder: A randomized controlled study

**DOI:** 10.1002/ped4.70058

**Published:** 2026-04-21

**Authors:** Yanru Liu, Wanling Zhang, Xuejiao Sun, Xiaoxuan Fan, Na Hu, Xuanzi Zhou, Xiao Leng, Ying Li

**Affiliations:** ^1^ Department of Psychosomatic Medicine Beijing Children's Hospital, Capital Medical University, National Center for Children's Health Beijing China; ^2^ Department of Clinical Psychology Beijing Huilongguan Hospital Beijing China; ^3^ Department of Clinical Psychology Fengtai Maternal and Child Health Hospital Beijing China

**Keywords:** Adolescents, Anxiety symptoms, Dance movement therapy, Depressive symptoms, Major depressive disorder, Self‐esteem

## Abstract

**Importance:**

Major depressive disorder (MDD) significantly impairs psychological well‐being and social functioning in adolescents. While pharmacotherapy is the most commonly used first‐line treatment in current clinical practice, its limitations highlight the need for complementary approaches.

**Objective:**

This study aimed to compare dance movement therapy (DMT) combined with pharmacotherapy to pharmacotherapy alone in terms of their effects on the severity of depressive symptoms, anxiety symptoms, and self‐esteem in adolescents with MDD.

**Methods:**

Eighty adolescents with MDD from Beijing Huilongguan Hospital were randomly assigned to either an intervention group (DMT and pharmacotherapy, *n* = 40) or a control group (pharmacotherapy alone, *n* = 40). Participants completed the Hamilton Depression Rating Scale, Hamilton Anxiety Rating Scale, and Rosenberg Self‐Esteem Scale at baseline and after the 6‐week intervention period.

**Results:**

For depressive symptoms, repeated‐measures analyses of variance revealed a significant main effect of time, *F* (1, 78) = 180.50, *P* < 0.001, partial *η* ^2^ = 0.698, as well as a significant time × group interaction, *F* (1, 78) = 17.20, *P* < 0.001, partial *η* ^2^ = 0.181. For both anxiety symptoms and self‐esteem, a significant main effect of time was observed, but the time × group interaction was not significant.

**Interpretation:**

DMT combined with pharmacotherapy improved depression symptoms more than pharmacotherapy alone, but this enhanced effect was not observed for anxiety symptoms or self‐esteem.

## Graphical abstract

The current study was among the first clinical trials that combined dance movement therapy (DMT) and pharmacotherapy to enhance self‐esteem and alleviate anxious and depressive symptoms in adolescents with major depressive disorder. Results indicated that DMT combined with pharmacotherapy improved depression symptoms more than pharmacotherapy alone, but this enhanced effect was not shown for anxiety symptoms or self‐esteem.

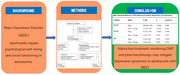

## INTRODUCTION

Major depressive disorder (MDD) adversely affects psychological health in adolescents, potentially engendering social and educational impairments.^1^ The core symptoms of MDD in adolescents are depressive symptoms, which are often accompanied by anxiety symptoms; together, these symptoms impair adolescents’ psychological functioning.^2^ Adolescent MDD often manifests as decreased self‐esteem, which may lead to social withdrawal.^3^ According to the World Health Organization, MDD is a leading cause of disability and illness among adolescents.^4^ An epidemiological survey showed that the overall prevalence of mental disorders among Chinese children and adolescents aged 6–16 years has reached a staggering 17.5%.^5^ Among these disorders, MDD accounts for 3.0%.^5^ In China, the prevalence of adolescent MDD has been rising, with surveys conducted in 2019 reporting that over 20% of Chinese adolescents exhibit depressive symptoms.^6^ This high prevalence presents significant challenges for mental health professionals, emphasizing the need for effective intervention strategies.^7^

Current interventions for adolescent MDD may be classified into pharmacological and non‐pharmacological approaches.^8^ The recommended pharmacological treatment for adolescent MDD is the use of selective serotonin reuptake inhibitors, with commonly used medications including fluoxetine, sertraline, and escitalopram. Non‐pharmacological treatments include psychotherapy, physical therapy, and complementary therapies. Among these, psychotherapy methods include cognitive‐behavioral therapy, adolescent interpersonal psychotherapy, family therapy, problem‐solving therapy, and supportive psychotherapy. Physical therapies include repetitive transcranial magnetic stimulation and modified electroconvulsive therapy. Complementary therapies include nutritional supplements and exercise.^9^ Although widely considered to be fundamental, pharmacotherapy's efficacy has been reported to be suboptimal for many adolescents, among whom risks of adverse events and suicide increased.^10^ Adverse drug reactions may potentially lead to treatment discontinuation.^11^ Poor medication adherence among adolescents further limits its efficacy.^12^ Studies have shown that the combination of pharmacotherapy and psychotherapy is more effective than using psychotherapy or pharmacotherapy alone.^13^, ^14^ Therefore, in practice, effective non‐pharmacological treatments urgently need to be combined with traditional pharmacotherapy. Studies have shown that the combination of mindfulness‐based cognitive therapy and pharmacotherapy may help reduce the recurrence of MDD and improve depressive symptoms during each episode.^15^ Additionally, exercise therapy and progressive muscle relaxation interventions can effectively reduce depressive symptoms in patients.^16^ Theoretically, integrating multiple treatment methods may provide a patient‐centered model, addressing both the biological and psycho‐social aspects of MDD.^17^

Dance movement therapy (DMT) is a psychotherapy method that uses dance or improvisational movement to express emotions and relieve stress, and can be implemented through both individual and group formats.^18^ Studies have shown that DMT reduces depressive symptoms, enhances quality of life, and improves social skills.^19^, ^20^ Studies have already demonstrated that DMT generally exerts positive effects in individuals with mild depression.^21^ Boon‐Soon^22^ implemented DMT among Latin American migrant adolescents, demonstrating that DMT significantly alleviated depressive symptoms in this population. Potential desirable effects of DMT include providing a non‐verbal means of emotional expression, enhancing body awareness and social support networks, and promoting the release of endorphins, thereby improving overall mood.^19^, ^23^, ^24^ From a developmental perspective, the critical period of adolescence highlights a significant need for expressive therapies such as DMT, which directly enhance participants’ creative exploration and psycho‐social development.^25^ Experimental evidence supporting DMT's effectiveness among adolescents with MDD requires broader validation.

The present study aimed to compare the effects of DMT combined with pharmacotherapy versus pharmacotherapy alone on the severity of depressive symptoms, anxiety symptoms, and self‐esteem in adolescents with MDD, thereby providing evidence‐based support for clinical intervention strategies.

## METHODS

### Ethical approval

This study was approved by the Ethics Committee of Beijing Huilongguan Hospital (Approval No.: 20220424). Written informed consent was obtained from all participants and their guardians.

### Participants

**FIGURE 1:**
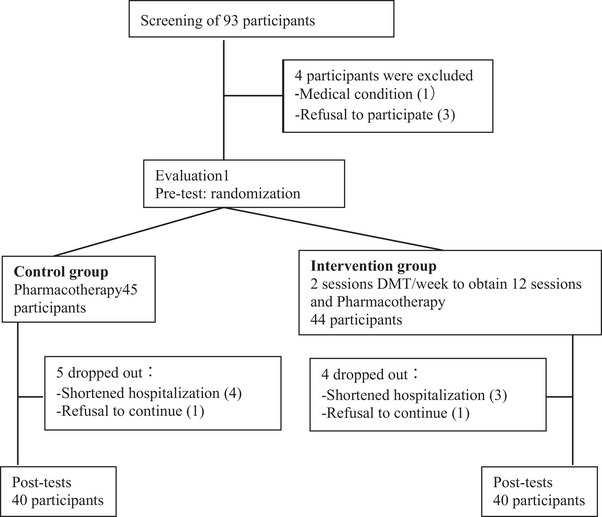
Flow chart of study design. DMT, dance movement therapy.

Adolescents with MDD were recruited from the inpatient department of Beijing Huilongguan Hospital between April 2022 and December 2023. A total of 93 participants were invited to participate, of which four were excluded (one due to a medical condition and three because of refusal to participate). Eighty‐nine participants were successfully enrolled and randomly assigned to the intervention group (treated with both DMT and pharmacotherapy) or the control group (treated with pharmacotherapy alone). Inclusion criteria were as follows: ‌1) met the diagnostic criteria for MDD according to DSM‐5; 2) aged 12–18 years; 3) had attended primary school and were able to read; and 4) voluntarily participated in the study. Exclusion criteria were: 1) history of electroconvulsive therapy within 6 months prior to enrollment; 2) history of systematic psychotherapy within 3 months prior to enrollment; 3) presence of suicidal or impulsive tendencies; and 4) presence of severe physical illness. Participants who met the inclusion criteria were randomly assigned to the intervention group or the control group at a 1:1 ratio using simple randomization (computer‐generated random numbers).^26^ There were 44 participants in the intervention group, with four dropouts (three due to shortened hospitalization and one because of refusal to participate), and 45 in the control group, with five dropouts (four due to shortened hospitalization and one because of refusal to participate). Ultimately, 40 participants in each group completed the study (Figure 1).

### Interventions

The participants voluntarily participated and signed an informed consent form, agreeing to complete the assessments. On each working day, all participants met with a psychiatrist twice daily, with each session lasting 20 min. Over the course of the study, each participant received a total of 60 psychiatric ward rounds. At the time of enrollment, participants in both groups had been maintained on stable medication doses for at least 2 weeks. Each participant was treated with a single selective serotonin reuptake inhibitor, including sertraline (50–200 mg/d), escitalopram oxalate (5–20 mg/d), or fluoxetine (20–60 mg/d). A subset of participants received adjunctive low‐dose atypical antipsychotics, including aripiprazole (5–10 mg/d) or quetiapine fumarate (25–50 mg/d), which included four of 44 participants in the intervention group and four of 45 participants in the control group. All medications were systematically recorded. During the study period, participants who experienced any changes in their medication regimens were excluded. However, no medication adjustments occurred during the study, and therefore, no participants were excluded for this reason. Participants in both groups received identical routine nursing care, and none received group psychotherapy or social work services.

DMT was administered twice a week for 60 min per session over a 6‐week period, totaling 12 sessions. The intervention comprised the following components: 1) Facilitators: Each session was led by a certified dance movement therapist, assisted by a psychiatrist. The dance movement therapist underwent over 750 hours of theoretical learning, clinical practice, and personal supervision related to dance movement therapy before obtaining the qualification of a registered dance movement therapist. 2) Therapy procedure: Stage 1: Warm‐up activities were designed to awaken body awareness, starting with light movements from various parts of the body, such as moving the hands, arms, face, neck, torso, and legs; Stage 2: Experiential movement exercises were conducted, such as exploring the difference between body tension and relaxation; Stage 3: Guided imagery and improvisational performance were introduced; for example, by playing soft, rhythmically strong instrumental music without lyrics,^27^ during which participants freely danced in the space, exploring their body sensations. Stage 4: Therapist assessment and feedback; for example, when a participant reported inability to move while listening to fast‐paced music and experienced anxiety, the therapist assessed this as fear and suggested body shaking techniques for release. 3) Therapeutic themes: The 12 sessions were divided into three thematic phases: body awareness and self‐perception (e.g., observing and touching one's hands, patting the arms while noticing internal feelings); emotional release (e.g., using a towel to hit a chair to express anger, or patting the body to relieve tension); and social skills training (e.g., pairing up and connecting two index fingers with a chopstick, then moving together to train teamwork and coordination).

### Data collection and assessment tools

#### Baseline information

We collected participants’ general demographic information, including age, gender, and years of education, as well as clinical data such as illness duration within 1 week before randomization.

#### Psychological assessment scales

Participants completed the Hamilton Depression Rating Scale (HAMD), Hamilton Anxiety Rating Scale (HAMA), and Rosenberg Self‐Esteem Scale at baseline and after the 6‐week intervention period. Given that participants met the criteria for MDD, depressive symptoms (i.e., scores on the HAMD) were considered the primary outcome. In view of the recognized importance of anxiety symptoms and self‐esteem for adolescent mental health and social functioning, these (i.e., scores on the HAMA and Self‐Esteem Scale [SES]) were treated as secondary outcomes.

HAMD was developed by Hamilton in 1960 to assess the severity of depression.^28^ The Chinese version of the HAMD scale showed internal consistency with a Cronbach's α coefficient of 0.714. The overall parallel validity and construct validity (as assessed through principal component analysis) were found to be satisfactory.^29^

HAMA was developed by Hamilton in 1959 to assess the severity of anxiety.^28^ The reliability coefficient (*r*) of the total score of the Chinese version of the HAMA scale was 0.93, while the reliability coefficients for individual symptom scores ranged from 0.83 to 1.00.^30^

SES was developed by Rosenberg in 1965. It includes two dimensions: self‐affirmation and self‐denial. Higher scores indicate higher levels of self‐esteem.^28^ The Cronbach's α coefficient of the Chinese version of the Rosenberg Self‐Esteem Scale was above 0.83, and the test‐retest reliability coefficient was 0.76. The scale had a good structural validity.^31^

### Sample size calculation

This study adopted “changes in depressive symptoms” as the primary outcome. The pilot study included 30 participants, with 15 participants in the intervention group (DMT and pharmacotherapy) and 15 participants in the control group (pharmacotherapy alone). According to the pilot data, the mean difference in HAMD scores before and after 6 weeks of intervention was 18.67 ± 6.89 in the intervention group and 9.20 ± 7.96 in the control group. With *α* = 0.05, *β* = 0.1, and a two‐tailed test, and assuming a 20% dropout rate, the sample size was calculated using the “Two‐Sample T‐Tests Allowing Unequal Variance” module in PASS 2021. The result indicated that at least 19 participants were needed per group, for a total of at least 38 participants. Based on the center's practical conditions, the planned sample size was 40 participants per group, totaling 80 participants.

### Data analyses

Data were analyzed using SPSS 25.0 software. Normality of continuous variables, including age, years of education, duration of illness, SES, HAMA, and HAMD scores, was assessed using the Shapiro–Wilk test, and all variables were found to follow a normal distribution. These variables were expressed as mean ± standard deviation. Categorical variables, including gender, ethnicity, residence, age at first onset, and medication status, were expressed as frequencies and percentages, with comparisons made using chi‐square tests. Repeated‐measures analysis of variance (RM ANOVA) was conducted to examine changes in depression (HAMD), anxiety (HAMA), and self‐esteem (SES) over time and between the two groups. All statistical tests were conducted using two‐tailed tests, and a *P*‐value of < 0.05 was considered statistically significant.

## RESULTS

### Demographics, clinical characteristics, and pre‐treatment scores for both groups

The intervention group consisted of 9 males and 31 females, aged 12–18 years (14.65 ± 1.63 years), with 6–12 years of education (8.30 ± 1.70 years), and a duration of illness ranging from 11 to 72 months (29.85 ± 15.85 months). The control group comprised 8 males and 32 females, aged 12–18 years (14.83 ± 1.58 years), with 6–12 years of education (8.48 ± 1.54 years), and a duration of illness ranging from 4 to 84 months (27.33 ± 17.69 months).

No significant differences were found in any demographic data (including gender, age, ethnicity, residence, and years of education) and clinical characteristics (duration of illness, first episode, and regular medication use) between the intervention and control group (all *P* > 0.05).

Before treatment, the intervention group had a HAMD score of 29.60 ± 10.55, a HAMA score of 18.80 ± 10.03, and a SES score of 21.70 ± 6.03. The control group had a HAMD score of 30.25 ± 11.88, a HAMA score of 20.20 ± 11.06, and a SES score of 20.70 ± 7.23. No significant differences were observed in the HAMD, HAMA, or SES scores between the two groups at baseline (*P* > 0.05), indicating comparability (Table 1).

**TABLE 1:** Demographics, clinical characteristics, and pre‐treatment scores for both groups

Variable	Intervention group (*n* = 40)	Control group (*n* = 40)	*t*/χ^2^	*P*
Gender (Male/Female)	9/31	8/32	0.08	1.000
Age (Years)	14.65 ± 1.63	14.83 ± 1.58	0.49	0.627
Ethnicity (Han/Other)	39/1	38/2	0.35	1.000
Place of residence (Urban/Rural)	34/6	37/3	1.13	0.481
Years of education	8.30 ± 1.70	8.48 ± 1.54	0.48	0.630
Duration of illness (Months)	29.85 ± 15.85	27.33 ± 17.69	−0.67	0.503
First episode (Yes/No)	33/7	36/4	0.95	0.518
Regular medication (Yes/No)	28/12	25/15	0.50	0.637
HAMD	29.60 ± 10.55	30.25 ± 11.88	−0.26	0.797
HAMA	18.80 ± 10.03	20.20 ± 11.06	−0.59	0.555
SES	21.70 ± 6.03	20.70 ± 7.23	0.67	0.503

Data are presented as mean ± standard deviation or *n*.

HAMA, Hamilton Anxiety Rating Scale; HAMD, Hamilton Depression Rating Scale; SES, Self‐Esteem Scale.

### Effects of DMT combined with pharmacotherapy on clinical outcomes

RM ANOVA was conducted to examine changes in depression (HAMD), anxiety (HAMA), and self‐esteem (SES) over time and between the two treatment groups. For depressive symptoms, RM ANOVA revealed a significant main effect of time [*F* (1, 78) = 180.50, *P* < 0.001, partial *η* ^2^ = 0.698] as well as a significant time × group interaction [*F* (1, 78) = 17.20, *P* < 0.001, partial *η* ^2^ = 0.181]. This interaction indicated that participants receiving DMT combined with pharmacotherapy exhibited significantly greater reductions in depressive symptoms over time than those receiving pharmacotherapy alone.

For anxiety symptoms, a significant main effect of time was observed [*F* (1, 78) = 51.51, *P* < 0.001, partial *η* ^2^ = 0.398], indicating a significant reduction in anxiety across both groups. However, the time × group interaction was not significant [*F* (1, 78) = 1.64, *P* = 0.204, partial *η* ^2^ = 0.021], suggesting that the addition of DMT did not result in greater improvement in anxiety compared with pharmacotherapy alone.

With respect to self‐esteem, a significant main effect of time was found [*F* (1, 78) = 9.28, *P* = 0.003, partial *η* ^2^ = 0.106], reflecting overall improvement across both groups. The time × group interaction was not significant [*F* (1, 78) = 0.29, *P* = 0.591, partial *η* ^2^ = 0.004], indicating that improvements in self‐esteem did not differ significantly between the two treatment conditions (Table 2).

**TABLE 2:** Repeated‐measures analysis of variance (ANOVA) results for anxiety, depression, and self‐esteem

Variable	Effect	*F* (df)	*P*	Partial *η* ^2^
HAMD	Time	180.50 (1, 78)	<0.001	0.698
	Time × Group	17.20 (1, 78)	<0.001	0.181
HAMA	Time	51.51 (1, 78)	<0.001	0.398
	Time × Group	1.64 (1, 78)	0.204	0.021
SES	Time	9.28 (1, 78)	0.003	0.106
	Time × Group	0.29 (1, 78)	0.591	0.004

Repeated‐measures analysis of variance (RM ANOVA) demonstrated significant main effects of time for depression (HAMD), anxiety (HAMA), and self‐esteem (SES) (all *P* < 0.01). A significant time × group interaction was observed only for depressive symptoms, *F* (1, 78) = 17.20, *P* < 0.001, partial *η* ^2^ = 0.181, indicating greater improvement in the DMT combined with pharmacotherapy group compared with pharmacotherapy alone. No significant time × group interactions were found for anxiety or self‐esteem (both *P* > 0.05).

### Covariate analyses

To examine whether baseline demographic and clinical characteristics influenced treatment‐related changes in outcomes, additional multivariate analyses were conducted, controlling for age, years of education, duration of illness, sex, ethnicity, residence, first episode status, and regular medication use. As shown in Table S1, no significant main effects of group were observed for depression, anxiety, or self‐esteem after adjusting for these covariates (all *P* > 0.05). Furthermore, no significant interaction effects were found between group and any demographic or clinical variables, and all effect sizes were small (all partial *η* ^2^ < 0.05).

## DISCUSSION

The current study was among the first clinical trials to combine DMT and pharmacotherapy to enhance self‐esteem and alleviate anxious and depressive symptoms in adolescents with MDD. The results indicated that DMT, when combined with pharmacotherapy, yielded greater improvement in depressive symptoms than pharmacotherapy alone. However, this enhanced effect was not observed for anxiety symptoms or self‐esteem.

Consistent with findings of numerous existing studies, DMT proved to be effective in treating MDD. For example, Ptak and Szyc's analysis of existing studies demonstrated that DMT and other dance‐based interventions significantly reduce depressive symptoms and enhance psychological well‐being, with the structured format of DMT yielding more consistent and pronounced benefits.^21^ Jeong et al.^24^ implemented DMT in a cohort of 40 middle school students over a 12‐week intervention period and demonstrated its efficacy in improving emotional states. Karkou et al.^32^ conducted a meta‐analysis of adult depression studies with subgroup and sensitivity analyses, and concluded that moderate‐ to high‐quality evidence supports the effectiveness of DMT in reducing depressive symptoms. Additionally, Koch et al.^19^ reported that dance therapy alleviated depression and anxiety while improving quality of life, as well as interpersonal and cognitive skills. Conducted in group settings, DMT encouraged social interaction and support network development, which is crucial for MDD recovery.^33^ As a physical activity, dancing was also found to stimulate endorphin release, thereby improving mood and mental well‐being.^34^ Potential mechanisms underlying the antidepressant effects of DMT may include providing a safe space for adolescents to express internal emotions through bodily movement. This non‐verbal expression facilitates emotional release, thereby reducing depressive symptomatology.^19^, ^24^ Future research is warranted to further elucidate and expand upon these underlying mechanisms.

Although both interventions significantly reduced anxiety symptoms, DMT did not provide additional benefits beyond pharmacotherapy alone for this outcome. Improvements in self‐esteem were observed over time in both groups; however, no significant time × group interaction was detected, indicating comparable effects between the two interventions. These findings were inconsistent with previous research. One study applied DMT to a group of adolescent girls, resulting in a positive impact on self‐esteem.^35^ A meta‐analysis of 41 controlled intervention studies (*n* = 2374, from January 2012 to March 2018) found that DMT significantly reduced anxiety levels.^19^ These findings supported the potential effectiveness of combining DMT and pharmacotherapy for improving self‐esteem and reducing anxiety. Dancing enhanced body awareness and self‐esteem,^19^ enabling patients with MDD to better perceive and understand their emotions and physical states, thereby promoting emotional regulation.^33^, ^36^ This discrepancy may be attributable to the fact that our participants were inpatients. In clinical settings, inpatients often present with more severe depressive symptoms, which may mask variations in anxiety and self‐esteem. Future studies should include larger sample sizes and further validate these findings in outpatient populations with mild to moderate depression.

Our study has several limitations. First, the relatively small sample from a single center limits statistical power and generalizability. Second, dance is a form of body language. During DMT, becoming aware of one's bodily movements helps in recognizing emotional changes and, in turn, using physical movement to regulate emotions. But during the implementation of DMT, we failed to put emphasis on conscious awareness of movement connectivity. Previous studies have demonstrated that muscle relaxation can improve mood.^37^ Incorporating exercises such as shoulder and neck relaxation and finger joint mobility might be a useful improvement in future studies. Combining mindfulness interventions may also help enhance self‐esteem and reduce anxiety symptoms.^38^ Third, the 6‐week intervention period may have been insufficient to observe significant changes in self‐esteem and anxiety. Future studies should extend the intervention and follow‐up periods to evaluate the long‐term effects of DMT.^36^ Finally, self‐reported scales may introduce bias from social desirability and transient emotional states. Future research should incorporate objective indicators (e.g., physiological and behavioral measures), alongside participants’ subjective evaluations (e.g., whether they perceive the therapy as effective in improving their condition), to provide a more comprehensive evaluation. The high completion rate of follow‐up assessments strengthens the generalizability of our findings to the target population. Despite these limitations, our study provides evidence for potential non‐pharmacological interventions for adolescents with depression, and further in‐depth research is warranted.

Additionally, this study offers evidence for mental health professionals to incorporate expressive therapies into standard treatment protocols. It highlighted the importance of considering patients’ individual needs and adopting a more comprehensive treatment approach when addressing adolescent MDD.^39^

In conclusion, DMT combined with pharmacotherapy improved depression symptoms more than pharmacotherapy alone; however, this enhanced effect was not observed for anxiety symptoms or self‐esteem. Therefore, DMT may be a valuable adjunctive treatment for adolescents with MDD receiving pharmacotherapy. Future research should address the limitations of this study and further explore the long‐term effects and mechanisms of DMT.

## CONFLICT OF INTEREST

None.

## Supporting information



Supporting Information
