# Supplementary material for: Combining dance movement therapy and pharmacotherapy in adolescents with major depressive disorder: A randomized controlled study
Source: Pediatr Investig. 2026 Apr 21:10.1002/ped4.70058. Online ahead of print. doi: 10.1002/ped4.70058 (PMC13398827; doi:10.1002/ped4.70058)
Supplement: Supplementary file 1 — Supporting Information [file PED4-9999-0-s001.pdf]

Supplementary Material for

Combining dance movement therapy and pharmacotherapy in adolescents with major depressive disorder: A randomized controlled study

Yanru Liu, Wanling Zhang, Xuejiao Sun, Xiaoxuan Fan, Na Hu, Xuanzi Zhou, Xiao Leng, Ying Li

Table S1 Multivariate tests of covariates and interaction effects on depression, anxiety, and self-esteem

Dependent Variable	Effect	Wilks' Lambda	*F*	*df* (Hypothesis)	*df* (Error)	*P*	Partial η^2
**Depression**	Group	0.984	0.926	1	57	0.34	0.016
	Age	1	0.007	1	57	0.93	<0.001
	Years of education	1	0.008	1	57	0.93	<0.001
	Duration of illness	0.999	0.061	1	57	0.81	0.001
	Sex	0.995	0.287	1	57	0.59	0.005
	Ethnicity	0.997	0.159	1	57	0.69	0.003
	Residence	0.999	0.034	1	57	0.85	0.001
	First episode	0.998	0.121	1	57	0.73	0.002
	Regular medication	0.998	0.111	1	57	0.74	0.002
	Group × Sex	0.965	2.065	1	57	0.16	0.035
**Anxiety**	Group	0.984	0.926	1	57	0.34	0.016
	Age	1	0.007	1	57	0.93	<0.001
	Years of education	1	0.008	1	57	0.93	<0.001
	Duration of illness	0.999	0.061	1	57	0.81	0.001
	Sex	0.995	0.287	1	57	0.59	0.005
	Ethnicity	0.997	0.159	1	57	0.69	0.003
	Residence	0.999	0.034	1	57	0.85	0.001
	First episode	0.998	0.121	1	57	0.73	0.002
	Regular medication	0.998	0.111	1	57	0.74	0.002
	Group × Sex	0.965	2.065	1	57	0.16	0.035

**Self-esteem (SES)**	Group	1	0.002	1	57	0.97	<0.001
	Age	0.999	0.082	1	57	0.78	0.001
	Years of education	0.997	0.197	1	57	0.66	0.003
	Duration of illness	0.962	2.246	1	57	0.14	0.038
	Sex	0.990	0.57	1	57	0.45	0.010
	Ethnicity	0.984	0.902	1	57	0.35	0.016
	Residence	1	0	1	57	0.98	<0.001
	First episode	1	0.01	1	57	0.92	<0.001
	Regular medication	0.979	1.219	1	57	0.27	0.021

Note: Only main effects and selected two-way interaction effects are presented. Higher-order interaction effects were not statistically significant and are therefore not shown. All tests were based on Wilks' Lambda.
